# Spontaneous preference for unpredictability in the temporal contingencies between agents' motion in naive domestic chicks

**DOI:** 10.1098/rspb.2022.1622

**Published:** 2022-11-09

**Authors:** Bastien S. Lemaire, Orsola Rosa-Salva, Margherita Fraja, Elena Lorenzi, Giorgio Vallortigara

**Affiliations:** Center for Mind/Brain Sciences, University of Trento, Piazza Manifattura, 1, 38068 Rovereto, TN, Italy

**Keywords:** motion perception, domestic chicks, animate agents, social predispositions

## Abstract

The ability to recognize animate agents based on their motion has been investigated in humans and animals alike. When the movements of multiple objects are interdependent, humans perceive the presence of social interactions and goal-directed behaviours. Here, we investigated how visually naive domestic chicks respond to agents whose motion was reciprocally contingent in space and time (i.e. the time and direction of motion of one object can be predicted from the time and direction of motion of another object). We presented a ‘social aggregation’ stimulus, in which three smaller discs repeatedly converged towards a bigger disc, moving in a manner resembling a mother hen and chicks (versus a control stimulus lacking such interactions). Remarkably, chicks preferred stimuli in which the timing of the motion of one object could not be predicted by that of other objects. This is the first demonstration of a sensitivity to the temporal relationships between the motion of different objects in naive animals, a trait that could be at the basis of the development of the perception of social interaction and goal-directed behaviours.

## 1. Introduction

Motion perception is fundamental to orient in the visual environment. For instance, it allows detection of the presence of other living animals, provides insights into their internal states and helps to anticipate their behaviours [1,2]. The detection of animate agents (other living animals) can be driven by very simple motion cues. Intriguingly, similar cues capture visual attention in humans [3] and non-human animals, such as domestic chicks [1,4]. Studies on newly hatched chicks, a precocial species that can be tested immediately after hatching, before any visual experience, have been used to reveal inborn predispositions to attend to social partners [4–6]. Visually naive chicks are spontaneously drawn toward motion properties typical of animate creatures [1,5]. Intriguingly, often these same motion properties also attract human infants and are perceived by human adults as associated with the presence of animate agents (for reviews see [4,7]).

For instance, humans detect moving objects faster than stationary ones [8] and categorize them as more animate [9]. Similarly, naive chicks preferentially approach fast-moving objects rather than stationary or slower ones (see [10] and [11] for opposite results). Although speed seems to provide information on the presence of animate agents, inanimate objects can move at considerable speed, if propelled by external forces. A distinguishing feature of animate agents is self-propulsion. This is the ability of an agent to change its own state, such as starting to move from rest, changing speed and changing direction. Self-propulsion implies that an object has an internal source of energy, which inanimate objects lack, in the natural environment. Indeed, human adults attribute higher animacy rankings to objects that change speed and direction [12]. Human newborns preferentially look to an agent that starts to move on its own and changes speed in comparison to an agent moving at a constant speed [13,14]. Similarly, visually naive chicks preferentially imprint on an agent that starts to move on its own [15] and prefer to approach agents that spontaneously alter their speed while in movement [16–18]. These results suggest inborn predispositions shared across taxa, triggered by elementary motion cues associated with self-propulsion.

Besides self-propulsion, the way animals move is impacted by their body structure. This, too, can be used as a cue to recognize animate agents. For example, most animals keep their main-body axis aligned with their motion direction. Human adults consider agents moving in a such a way as more animate than others that do not ([12] see also [19] for evidence of human infants' assumptions on how animate agents' body structure impacts their behaviour). Agents that maintain their body axis aligned with their motion trajectory are also spontaneously preferred by naive chicks [20]. Moreover, because of the constraints posed by their skeletal structure, animals move in a semi-rigid manner (some parts of their bodies always maintain fixed distance from each other, e.g. wrist and elbow, while other parts can vary their distance according to the animal's movement). This feature is conveyed very well when biological motion patterns are presented through point-light displays, in which only few dots placed on the limbs and joints of the moving animal are visible [21]. Human adults and infants, cats, spiders, fish and naive chicks are all attracted or strongly react to biological motion patterns [21–26]. Intriguingly, rather than focusing on whether the body structure resembles the body shape of a specific animal, naive chicks and newborn babies are generally attracted by the semi-rigid property of the motion pattern [26–28].

When multiple agents move within the same visual scene, further cues might be used to recognize animate agents. Different objects whose movements are reciprocally contingent in space and/or time (‘social contingency’ [29]) usually elicit the perception of social interactions and goal-directed behaviours in human observers (e.g. [30,31]). Some pioneering studies conducted in quails (another precocial galliform species) have shown that quail chicks show stronger imprinting for objects whose movements are temporally contingent with the chick's own behaviour ([32]; see also [33,34] for auditory imprinting). This is in line with evidence obtained in human infants. Infants seem to consider unfamiliar objects that react contingently to the infant's own behaviour as animate intentional agents (e.g. [35], see also [36,37]).

Intriguingly, adult dogs have shown a preference for an agent displaying contingent reactivity (consistent and predictable relations between its actions and the behaviour of another agent) [38]. Dogs seemed to consider an object as an animate agent, only if its movements are temporally contingent on the verbal command of a human. However, this effect was displayed only if the behaviour of the agent was perfectly predictable from the verbal commands. That is, agency was attributed to the object only if it responded to every single verbal command, although with a minimal and approximately constant delay, as in natural interactions. When the responses of the object to the verbal command became less predictable in terms of frequency or temporal delay, dogs did not seem to consider the object as an animate agent anymore.

A very prominent case, in which the spatio-temporal contingencies between the movements of different objects play a role in the recognition of animate agents, is that of chasing patterns. In these stimuli, a target moves around the screen and at least one chaser follows it, adapting its trajectory and velocity to the target's path, to reduce its distance from the target. Often, the target object reacts to the behaviour of the chaser, increasing its velocity or changing its motion direction when the chaser gets too close. During chasing, the behaviour of both agents is obviously contingent in space and time. In human adults, chasing displays are easily detected and are perceived as an interaction between animate agents performing and goal-directed intentional behaviours [30,31,39–43]. Intriguingly, some evidence suggests that, in the perception of chasing, the temporal contingency between the movement of the agents drives the perception of a social interaction, while the spatial contingencies allow recognition of the kind of interaction involved [31]. Some studies have also proposed that human observers immediately recognize the type of social interaction present in chasing stimuli and thus often direct their visual attention to explore stimuli with less predictable spatio-temporal contingencies [44,45]. In the human literature, chasing has thus often been used to test how the presence of spatio-temporal contingency between the movements of different objects affects the recognition of animate agents, of the presence of a social interaction and even the attribution of intentional goal-directed behaviour.

Human infants as young as three months can already discriminate between a chasing pattern and a random one and are attracted by the former ([46,47]; but see [42] for the developmental trajectory of this trait). By nine months of age, infants might already interpret chasing as a goal-directed behaviour [47] (see also [48]). This may indicate that recognition of the spatio-temporal contingencies defining chasing events is independent from enculturation. However, some studies have suggested that young infants might be strongly attracted by low-level properties of the chasing displays, such as the frequency of velocity changes in the agents' motion. On the contrary, the spatial contingency between the motion of the different agents on the screen would play a detectable, but minor, role in infants’ preferences [46]. Thus, the role of the spatio-temporal contingencies between the movement of different agents in determining early/inborn attention towards chasing patterns is still unclear.

Until now, only a few studies have been conducted on chasing stimuli in non-human animals, for instance by demonstrating that squirrel monkeys can be trained to discriminate chasing events from random motion patterns. Some monkeys could even spontaneously discriminate chasing from other motion patterns, in which the two objects show high spatio-temporal correlations, but do not depict a chasing interaction, indicating a potential sensitivity to the goal-directed nature of the depicted behaviour [49]. In a similar experiment, pigeons showed a limited, although significant, ability to learn to recognize a chasing-like stimulus [50]. Even more interestingly, dogs have shown a strong interest in chasing-like motion [51]. However, in subsequent studies, dogs showed a more complex pattern of results, depending also on the specific shape of the stimuli used. This included an initial preference for chasing or an initial absence of preference, followed by an increase in attention towards independent motion [44,45] (see also [52] for evidence of an opposite pattern in cats). Finally, in their most recent study, focusing on two specific dog breeds, Abdai *et al*. [53] did not find any clear-cut evidence of differential attention towards chasing stimuli, at least at the group level. Please note also that the preference for the independent motion is in contrast with the results of Tauzin *et al*. [38], which reported recognition of animate agents only in the presence of perfect reciprocal predictability of the agents' behaviours. Results in dogs thus seem to be partially contradictory or at least strongly influenced by procedural aspects.

Sensitivity to the spatio-temporal contingencies between the behaviour of different agents has never been tested so far in naive animals, despite this being a fundamental cue for the recognition of social interactions and goal-directed behaviours. Thus, it is still unclear whether the use of spatio-temporal contingencies between the motion of multiple agents, to detect the presence of living entities, is an inborn social predisposition or a trait that develops with experience. Moreover, the only evidence available in bird species indicated a limited sensitivity to the typical chasing patterns used for human experiments [50], while still suggesting some sensitivity to the coordinated converging motion of multiple objects. Thus, it is unclear whether recognition of chasing, and of the spatio-temporal contingencies that characterize it, can be found in this taxon (but see [54]; and [55] for pigeons’ ability to learn to discriminate stimuli based on the presence of elementary spatial contingencies between the motion of multiple objects).

Our aim was to investigate whether the sensitivity to ‘social contingencies' between the motion of animate agents engaged in an apparent social interaction is present before visual experience. To do so, we took advantage of chicks' precocity and inborn predispositions to approach animate agents. We investigated whether chicks would respond preferentially to agents whose motions were reciprocally contingent in space and time. Since chasing patterns have been widely used to study the perception of ‘social contingencies', we decided to include a form of chasing in our stimuli. However, we adapted the stimuli to the ecology and needs of our animal model (i.e. identifying the most appropriate imprinting object, keeping brood cohesion). To do so, we embedded these chasing sequences in the context of a social aggregation event, in which three smaller agents converged towards a bigger one. We discovered that chicks specifically attend to the temporal contingencies of the motion patterns. Moreover, we found that chicks spontaneously preferred stimuli (or situations) in which the timing of different agents’ motion was reciprocally unpredictable.

## 2. General methods

We conducted six experiments using the same procedure, but with different stimuli. The general procedure is described here below.

### (a) Subjects

Subjects were domestic chicks (*Gallus gallus domesticus*) of the Aviagen Ross 308 strain, obtained from a local commercial hatchery (Azienda Agricola Crescenti). The eggs were incubated at 37.7°C and 40% humidity and maintained in darkness. Three days before hatching, we moved the eggs into a hatching chamber (37.7°C and 60% humidity), where they hatched in complete darkness, to prevent any visual experience before the test. Each chick was tested only once. A new set of naive chicks was obtained for each experiment. Immediately after the test, the animals were housed in social groups within our animal house, in standard conditions optimal for this species, and then donated to local farmers.

### (b) Apparatus

**Figure 1.**
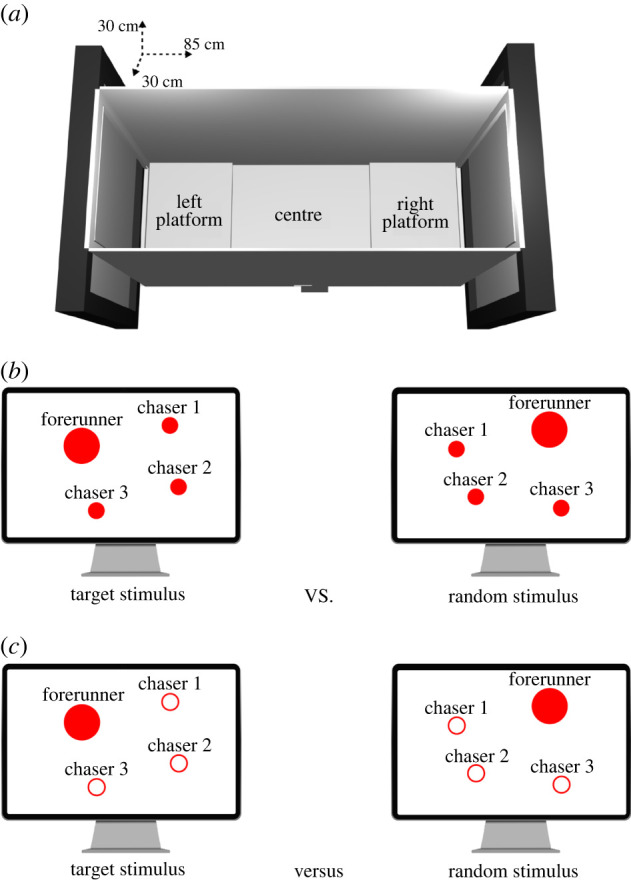
Representation of the test apparatus (*a*), the agents used to build the motion patterns in Experiments 1–3, 5–6 (*b*) and in Experiment 4 (*c*). (Online version in colour.)

We used the same testing apparatus as previous studies that investigated the spontaneous preferences of domestic chicks [16–18] (figure 1*a*). It consisted of a simple runway composed of a central zone and two lateral choice sectors. The central zone, equidistant from the two monitors, was 45 cm long. The two lateral sectors, adjacent to the two monitors, were each 20 cm long and were elevated by a platform 1.5 cm above the central sector. Each platform faced a high-frequency screen (Asus MG248QR, 120 Hz). A 30 × 30 cm portion of the monitor (in which the stimuli were played) was visible to the chicks through the opening at the end of the apparatus. To approach the stimuli playing on the monitors, the chicks had to climb on the corresponding platform. The only source of illumination to the apparatus was provided by the test stimuli.

Above the apparatus, a video camera recorded the animal's location within the test arena. This video was also feed to a screen located in the same room. This allowed the experimenter to manually code the time spent by the animal on each platform.

### (c) Stimuli

For each experiment, we tested chicks' choice between a ‘target stimulus’, for which a preference was expected, and a ‘control stimulus’. For instance, in our first experiment, the target stimulus depicted a social aggregation event, while a so-called random stimulus was used as control.

The stimuli were 30 s video clips manually generated with Blender 2.8 (and looped for the test duration of 6 min). Each stimulus depicted the motion of two kinds of agents: a forerunner (5 cm diameter) and three chasers (3 cm diameter each) (figure 1*b*). Each 30 s video clip was composed of eight scenes, each in turn composed of five sequences. In every sequence, the agents followed a different trajectory, to create a less repetitive stimulation.

**Figure 2.**
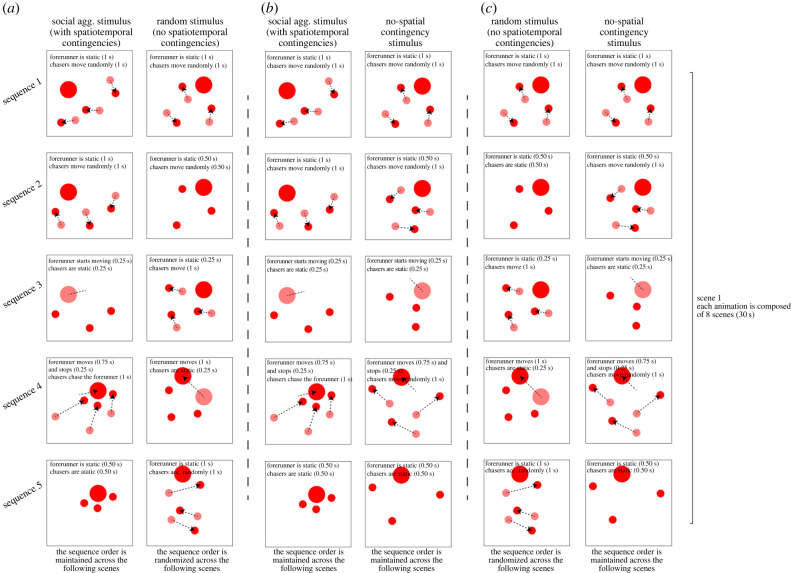
Graphical illustration of five motion sequences manually generated to create the stimuli for Experiment 1 (*a*), 5 (*b*) and 6 (*c*). For each animation generated, eight different scenes of five motion sequences were rendered. All the scenes of each stimulus followed the same structure defined by the five sequences, but the trajectory of the agents varied between each scene. We balanced the velocity and the distance travelled by the agents for each animation pair rendered. (Online version in colour.)

In the social aggregation stimulus, the forerunner had a simple motion pattern: it remained still for 2.75 s (e.g. sequence 1 and 2 on the left column of figure 2*a*) and then it moved along a straight line (in a randomly chosen direction) for 1 s (e.g. sequence 3 and 4 on the left column of figure 2*a*), where again it stopped for 2.75 s (e.g. sequence 5 on the left column of figure 2*a*) and so on (available at https://doi.org/10.6084/m9.figshare.20347401 or as electronic supplementary material).

As for the three chasers, their movements were always synchronized (i.e. they started/stopped to move all at the same time). However, each chaser had a different starting position and trajectory. In the social aggregation stimulus, the chasers moved according to the following structure. When the forerunner was still, each chaser was moving in a random direction (unrelated to the position of the forerunner and of the other chasers) for 1 s (sequence 1 and 2 on the left side of figure 2*a*). When the forerunner started to move, the chasers remained still for 0.25 s (sequence 3 on the left side of figure 2*a*). In the next sequence (sequence 4 on the left side of figure 2*a*), the chasers converged towards the position of the forerunner (this movement lasts 1 s). Finally (sequence 5 on the left side of figure 2*a*), the chasers remained still for 0.5 s in proximity of the forerunner. This movie was meant to reproduce a social aggregation event potentially happening in the natural environment of chicks, thus avoiding the perception of a predation event that might have induced aversive responses in chicks. The forerunner (bigger disc) was meant to represent the ‘mother hen’, while the chasers (smaller discs) were meant to represent ‘chicks’ that explore the surrounding environment when the mother remains still. Then, when the hen appears to be moving away, the ‘chicks’ converge back to her (social aggregation event) to maintain contact with the imprinting object and not to lose brood cohesion. This motion sequence also exploited the fact that birds might be particularly sensitive to convergent motion of multiple agents [50].

The random stimulus was created using the same basic ‘elements’ as the social aggregation stimulus. Each of the agents had the same basic motion properties as in the social aggregation stimulus, covering the same cumulative distance at the same velocity in the two displays. In the random stimulus too, the movements of the three chasers were synchronized, while their trajectories differed. Moreover, each agent showed periods of motion and of stillness of the same duration as in the social aggregation stimulus. For instance, in both stimuli, the forerunner was moving for periods of 1 s and being still for periods of 2.75 s. However, in the random stimulus, we randomized both the order of the various motion events and the trajectory followed by each agent. This disrupted the spatial and temporal contingencies between the motion of the different agents that defined the social aggregation events. To human observers, in the control stimuli, all the agents appeared to be moving randomly on the screen without being engaged in a recognizable form of social interaction.

In each experiment, we manipulated specific aspects of the stimuli, as detailed below (the videos used for each experiment are available at https://doi.org/10.6084/m9.figshare.20347401).

### (d) Procedure

The test was run on the first day after hatching. Each visually naive chick was taken from the incubator in complete darkness and carried in a closed opaque container to the experimental room. At the beginning of the test, the chick was placed in the central zone, facing one of the two long walls (the initial orientation of the chicks towards one or the other long wall and the left–right position of the target stimulus in the apparatus were counterbalanced between subjects). The test lasted for 6 min. During the test, when a chick entered one of the lateral sectors (by climbing on the corresponding platform), this was considered as a choice for the adjacent stimulus. In each experiment, we defined one stimulus (for which a preference was expected, usually the social aggregation stimulus) as a target stimulus, and the other stimulus as a control.

The dependent variables that we analysed were the same as in our previous studies (e.g. [16,20]). The first dependent variable recorded was the first choice, defined as the first stimulus approached by each animal during the test (by climbing on the corresponding platform): the target or the control stimulus. Moreover, we calculated the animals' preference for the target stimulus as percentage of time spent on the platform close to this stimulus, over the total choice time, using the following formula:

$$\begin{array}{rl} \text{preference for target stimulus}= & \frac{\text{time spent by the target stimulus}}{\text{time spent by the two stimuli}}\\ & \times 100. \end{array}$$

Values could range from 0% (full preference for the control stimulus), to 100% (full preference for the target stimulus), whereas 50% represented the absence of preference.

The time spent close to the stimuli was scored online as in Rosa-Salva *et al*. [16,20]. Afterwards, around 10% of the videos (25 randomly selected chicks) were re-coded by an experimenter blind to the position of the stimuli. The correlation between the percentage of preference for the target stimulus obtained in the online coding and in the offline blind coding confirmed the reliability of our initial coding (Pearson's correlation = 1, *p* < 0.001).

### (e) Statistical analysis

The sample size was estimated *a priori* using a power analysis [56] with an effect size (d) of 0.44 and an alpha of 0.05. This revealed that 45 individuals per experiment were required to achieve a power of 0.8 for a two-tailed Wilcoxon one-sample test against chance (50%), on the dependent variable percentage of preference for the target stimulus. The effect size was estimated based on preliminary observations conducted in our laboratories (unpublished). This *N* was rounded to 46 animals per experiment, to allow within-sample balancing of stimulus presentation side. Since a new sample of naive chicks was used for each experiment, 276 chicks were employed overall.

The data distribution normality was assessed by looking at the residuals' distribution (Q-Q plot) and Shapiro–Wilk tests. In all experiments, the residuals’ distributions violated the normality assumptions. Therefore, non-parametric tests were used.

To determine whether the animals expressed more frequently their first choice for the target stimulus or for the control stimulus we performed a chi-square test of independence.

To examine whether chicks had a significant preference for spending more time close to either stimulus, we performed one-sample Wilcoxon tests against chance level (50%). Moreover, for each experiment, we performed a permutation test with F-probabilities to investigate if there was any effect of sex on the animals' preference.

## 3. Experiment 1

This experiment aimed to investigate whether chicks have a spontaneous preference for a motion pattern that mimics social aggregation events and contains spatio-temporal contingencies reminiscent of chasing stimuli.

As described above, in the target stimulus, we represented social aggregation using two different kinds of agents. For the sake of simplicity, we will call the two kinds of agents ‘forerunner’ and ‘chasers’, even though our social aggregation stimuli do not represent prototypical chasing displays (figure 1*a*). The forerunner moves in a random direction from time to time. The three chasers move around when the forerunner is static and aggregate around the forerunner when it changes its position (left column of the figure 2*a*). Thus, the movements of one type of agent are predictive of the movement of the other kind of agent both in time and in space (i.e. the movements of the two types of agents are characterized by reciprocal spatio-temporal contingency).

In the random stimulus, all the agents were moving in random directions, and there was no spatio-temporal contingency between the movement of the two kinds of agents. In this stimulus, both the motion direction of the different agents and the temporal sequence of their movements were randomized (right column of the figure 2*a*). In other words, the forerunner actions did not appear to trigger the chasers' movements neither spatially nor temporally. Indeed, in the random stimulus, the motions of the chasers and of the forerunner were not contingent in space or time, which means that the movement of one type of agent is not predictive of the movement onset/direction of the other kind of agents.

### (a) Results and discussion

**Figure 3.**
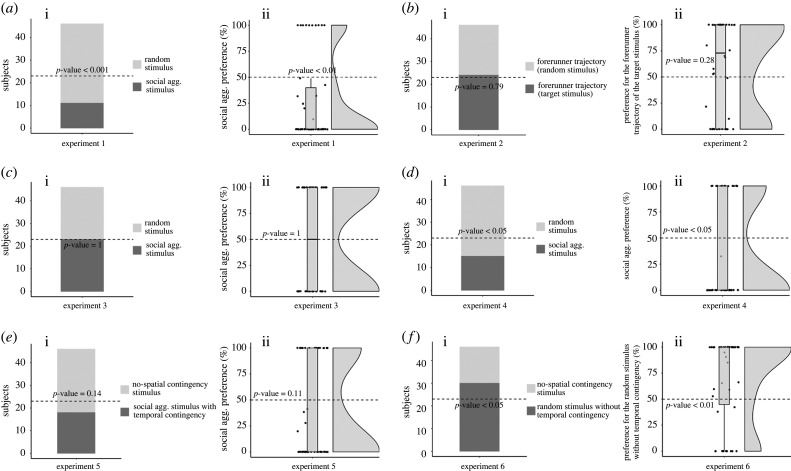
First stimulus approached by the subjects (i) and percentage preference for target stimulus (ii) for each experiment ((*a*) shows Experiment 1, (*b*) shows Experiment 2, (*c*) shows Experiment 3, (*d*) shows Experiment 4, (*e*) shows Experiment 5, (*f*) shows Experiment 6). On the percentage graphs, median and quartiles are shown in the box plot, while the dots represent the individual data and the cloud shows the data distribution.

No difference emerged between males and females with regard to the first stimulus approached (note that three subjects were not sexed because of a technical mistake and were thus not included in the analyses on sex effects, $\chi_{\left(1,43\right)}^{2}=0.02,$ *p* = 0.89). Thus, data of males and females were pooled for further analyses. Overall, the subjects preferentially approached the random stimulus (35 chicks approached the random stimulus, 11 chicks approached the social aggregation stimulus, $\chi_{\left(1,46\right)}^{2}=12.52,$ *p* < 0.001; figure 3*a*). Once again, the permutation test on the percentage of preference for the target stimulus did not reveal any effect of sex (*F*_1,41_ = 0.19, *p* = 0.89) on the animals’ preference. Thus, data of male and female subjects were pooled for the subsequent analysis. Also in this case, we observed a strong preference for the random stimulus (*V*_45_ = 270, *p* < 0.01, Cohen's *d* = 0.56, figure 3*a*), confirming the result of the other dependent variable.

Contrary to our initial expectations, in this experiment, we found a strong preference for the random stimulus. However, in our stimuli the trajectories of the forerunners and of the chasers were different between the two stimuli (even though features such as velocity, pauses and the distance travelled were matched between the stimuli). It could thus be possible that chicks' choice might actually reflect an idiosyncratic preference for the trajectory followed by either type of agent in the random stimulus. In the next two experiments, we tested whether the forerunners’ trajectories alone or the chasers' trajectories alone could drive the animals’ choice.

## 4. Experiment 2

In this second experiment, we investigated whether the forerunners' trajectories, when presented in isolation (without the chasers), could cause the animal preferences. We used the same animations as in the first experiment, but we removed the chasers from the scenes, so that only the two forerunners were visible.

### (a) Results and discussion

No difference emerged between males and females with regard to the first stimulus approached ($\chi_{\left(1,46\right)}^{2}=0.11,$ *p* = 0.74). Overall, the subjects approached both stimulis equally (24 chicks approached the target stimulus, 22 chicks approached the random stimulus, $\chi_{\left(1,46\right)}^{2}=0.09,$ *p* = 0.79; figure 3*b*). The permutation test on the percentage of preference for the target stimulus did not reveal any effect of sex (*F*_1,44_ = 0.05, *p* = 0.83). Also in this case, no preference was found between the two stimuli (*V*_45_ = 634.5, *p* = 0.28, Cohen's *d* = 0.16).

The results of this experiment clearly show that the forerunners movements alone did not cause the preference for the random stimulus observed in the first experiment.

## 5. Experiment 3

In this experiment, we tested whether an idiosyncratic preference for the trajectory of the chasers might drive the preference for the random stimulus. We used the same animations as in Experiment 1, but this time we removed the forerunners. Please note that, despite that, some sort of social aggregation events could be perceivable in the target stimulus. Indeed, even though the forerunner was not visible anymore, the chasers of the social aggregation stimulus still periodically converged towards its ‘position’, becoming closer to each other. The behaviour of the chasers of the target stimulus was thus both temporally linked (their movements were synchronized) and spatially linked (they periodically converged towards the same position). This allowed us to test whether agents whose behaviour is reciprocally contingent at both the spatial and the temporal level (chasers of the target animation) is preferred over that of agents whose behaviour is reciprocally contingent only at the temporal level (chasers of the random animation), while still following a less predictable sequence of movements.

### (a) Results and discussion

No difference emerged between males and females with regard to the first stimulus approached ($\chi_{\left(1,46\right)}^{2}=0,$ *p* = 1). Overall, the subjects approached both stimuli equally (23 chicks approached the chasers of the target stimulus, 23 chicks approached the chasers of the random stimulus, $\chi_{\left(1,46\right)}^{2}=0,$ *p* = 1; figure 3*c*). The permutation test on the percentage of preference for the chasers of the target stimulus did not reveal any effect of sex (*F*_1,4_ = 0.34, *p* = 0.56). Also in this case, no preference was found between the chasers of the two stimuli (*V*_45_ = 540.5, *p* = 1, Cohen's *d* = 0).

The results of this experiment showed that the movements of the chasers did not drive the preference observed in the first experiment. This suggests that something in the interaction between the two kinds of agents (forerunner and chasers) is necessary to direct the animals' attention toward the random stimulus. Moreover, chicks did not show any preference between chasers whose behaviour was reciprocally contingent only at the temporal level, and chasers whose behaviour was contingent both at the spatial and at the temporal level. This might suggest a minor role of spatial contingencies compared to temporal contingencies in chicks’ ability to recognize social interaction (a hypothesis that we further explored in Experiments 5 and 6).

## 6. Experiment 4

One alternative explanation to the results obtained in Experiment 1 could be that, rather than preferring the random stimulus, chicks avoided the social aggregation stimulus. This could be the case, for instance, if the social aggregation stimulus could be perceived as an aversive ‘predator-like’ stimulus. We designed our animations to avoid this possibility. However, perceptually, when the chasers aggregate around the forerunner, their movement could somehow create the perception of a looming object (since the configuration created by the chasers around the forerunner could be perceived as a ‘bigger red multi-component-object’ than the forerunner alone, giving the impression of the forerunner as a looming object). Since looming objects have been shown to reliably evoke fear responses in chicks [57], we tested this hypothesis in Experiment 4. Again, we used the same stimuli as in Experiment 1. However, in this experiment, the chasers were unfilled white circles, marked by a 2.5 mm red border, instead of full red discs (figure 1*c*). This manipulation should have strongly reduced any potential perception of looming created by the aggregation events. If chicks previously avoided the social aggregation stimulus, then the preference obtained in the Experiment 1 should disappear or at least be strongly reduced in Experiment 4.

### (a) Results and discussion

No difference emerged between males and females with regard to the first stimulus approached ($\chi_{\left(1,46\right)}^{2}=0.53,$ *p* = 0.47). Overall, the subjects preferentially approached the random stimulus (31 chicks approached the random stimulus, 15 chicks approached the social aggregation stimulus, $\chi_{\left(1,46\right)}^{2}=5.56,$ *p* < 0.05; figure 3*d*). The permutation test on the percentage of preference for the target stimulus did not reveal any effect of sex (*F*_1,44_ = 0.69, *p* = 0.41). Also in this case, overall, we observed a preference for the random stimulus (*V*_45_ = 360, *p* < 0.05, Cohen's *d* = 0.35).

In this experiment, we thus replicated the findings of the first experiment, while changing the perceptual features of the chasers, which stresses the robustness of our results. Moreover, in this experiment, we strongly reduced any potential looming effect, suggesting that this should not be the reason for chicks' preference for the random stimulus. This is also in line with the results of Experiment 3: even in that experiment, a looming effect—to a lower degree—could still be perceived in the target animation (when the chasers aggregated around the position of the missing forerunner). Nevertheless, the chicks did not show any avoidance of the social aggregation stimulus in Experiment 3.

The results of the first four experiments reveal that both the chasers and the forerunner need to be present in the visual scene to elicit the preference for the random stimulus over the social aggregation stimulus. Thus, some properties of the spatial and/or temporal contingencies defining the interaction between chasers and forerunner determine the preference for the random stimulus over the social aggregation stimulus. In Experiments 1 and 4, chicks showed a clear preference for the stimulus in which the behaviour of the forerunner and of the chasers did not show a clear reciprocal spatial or temporal interdependence. We hypothesize that this could be due to a preference for unpredictability in the spatial and or temporal contingencies between the movements of the chasers and of the forerunner.

## 7. Experiment 5

This experiment aimed to investigate whether the preference observed in Experiment 1 was influenced by the temporal contingency between different agents’ motions. Specifically, based on the results of Experiment 3, we wondered whether the absence of an evident temporal link between the behaviour of the forerunner and of the chasers attracted the chicks' attention and led them to approach the random stimulus. To test this hypothesis, we employed the same target animation (social aggregation stimulus) as in Experiment 1, testing it against a new control stimulus, henceforth called no-spatial-contingency stimulus. In this stimulus, at the spatial level, the movements of the chasers were still unrelated to the positions of the forerunner. That is, in the no-spatial-contingency stimulus the chasers never converged towards the position of the forerunner. The direction of their movements was always random (i.e. unrelated to the position of the forerunner, except to avoid collisions). However, the no-spatial-contingency stimulus was now matched to the social aggregation stimulus in the temporal contingencies between the movements of the chasers and of the forerunner (figure 2*b*). Thus, in both stimuli, the two kinds of agents followed the same rigid temporal sequence of motions (i.e. the forerunner was still for 1 s, during which the chasers are moving; movement of the forerunner followed, after 0.25 s, by movement of the chasers for 1 s, all agents still for 0.5 s and so on). To a human observer, this control stimulus could still give the impression that the movement of the forerunner triggered the movement of the chasers (i.e. some form of social interaction is happening between the two kinds of agents), even though no social aggregation is apparent in it.

### (a) Results and discussion

No difference emerged between males and females with regard to the first stimulus approached ($\chi_{\left(1,46\right)}^{2}=0.14,$ *p* = 0.71). Overall, the subjects approached both stimuli equally (18 chicks approached the social aggregation stimulus, 28 chicks approached the no-spatial-contingency stimulus, $\chi_{\left(1,46\right)}^{2}=2.18,$ *p* = 0.14; figure 3*e*). The permutation test on the percentage of preference for the trajectory of the target stimulus did not reveal any effect of sex (*F*_1,44_ = 0.14, *p* = 0.72). Also in this case, overall no preference was found between the two stimuli (*V*_45_ = 400, *p* = 0.11, Cohen's *d* = 0.24), confirming the result of the other dependent variable.

The absence of any significant preference between the two stimuli indicates that the spatial contingencies between the different agents' trajectories do not influence the animal's choice in this task. This supports the idea that the temporal contingencies between the agents' motion are driving chicks’ choices. Chicks could thus spontaneously prefer stimuli in which the timing of different agents' motion was reciprocally unpredictable.

## 8. Experiment 6

The aim of this experiment was to test if chicks are attracted by patterns in which the motions of different kinds of agents are not temporally contingent on each other (or at least not in a repetitive and predictable way). To do so, we tested the animals’ preference between the random stimulus used in Experiment 1 and the no-spatial-contingency stimulus developed for Experiment 5 (figure 2*c*). In this case, neither stimulus contained social aggregation events (there were no spatial contingencies between the motion direction of the two kinds of agents). However, the stimuli differed in the temporal contingency of their motion sequences. In the no-spatial-contingency stimulus, the agents' motion was temporally linked and followed the predictable structure described above. In the random stimulus, there was no evident temporal relationship between the movements of the chasers and of the forerunner.

### (a) Results and discussion

No difference emerged between males and females with regard to the first stimulus approached ($\chi_{\left(1,46\right)}^{2}=0,$ *p* = 1). Overall, the subjects preferentially approached the random stimulus used in Experiment 1 (30 chicks approached the random stimulus, 16 chicks approached the no-spatial-contingency stimulus, $\chi_{\left(1,46\right)}^{2}=4.26,$ *p* < 0.05; figure 3*f*). The permutation test on the percentage of preference for the random stimulus did not reveal any effect of sex (*F*_1,44_ = 0.03, *p* = 0.86). Overall, we again observed a strong preference for the random stimulus (*V*_45_ = 292, *p* < 0.01, Cohen's *d* = 0.50).

This experiment confirmed that chicks preferred the stimulus showing two kinds of agents of whose movements' timing was not reciprocally contingent (i.e. they preferred the stimulus in which the timing of the movement of one kind of agent could not be predicted by the movement of the other kind of agent).

## 9. Discussion

Over three different experiments, we demonstrated a strong and robust preference for the random stimulus over different versions of the social aggregation stimulus, in visually naive chicks. More specifically, we proved that this preference was driven by chicks’ attraction towards unpredictable temporal contingencies between the motion of different kinds of agents (Experiment 6). We were able to exclude alternative interpretations related to fear responses induced by looming like-stimuli (Experiments 4 and 3), or to idiosyncratic preferences for the trajectories of the forerunner or of the chasers (Experiment 2 and 3). This indicates a preference for stimuli in which the timing of the movement of one kind of agent cannot be predicted by the movement of the other kind of agent.

This finding is not in line with our initial expectations, nor with the results reported by [38], in which dogs seemed to consider as animate agents only objects that respond to the behaviour of a human in a highly predictable way (see also [51]; but see the subsequent work of the same research group for conflicting evidence, including a preference for random stimuli). In fact, based on the design of our stimuli and of chicks' motivation to approach appropriate social partners, we initially expected to find a preference for the social aggregation stimulus. Indeed, this stimulus represents a social interaction of potential ecological significance for chicks, in which the behaviour of multiple agents is reciprocally contingent (an important hallmark for the recognition of animate agents).

However, *a posteriori*, we can hypothesize that the temporal contingencies presented by the social aggregation stimulus might have been ‘too predictable’ for chicks. Indeed, the perception of naturalistic social interactions between animate agents might require imperfect spatio-temporal contingencies between the motion of different agents, such as slight delays between the actions of one agent and the response of the other (e.g. this has been theorized from a developmental perspective in [58]; see also [59]). For instance, objects moving in perfectly spatial and temporal synchrony (i.e. objects placed at different spatial locations, but simultaneously moving along identical trajectories) can disrupt animacy perception in human observers [60]. In line with that, in young bobwhite quail chicks, acoustical imprinting is facilitated by variable contingencies between the chicks' own vocalization and the auditory imprinting stimulus. However, in this experiment, ‘variable contingencies’ meant variations in the number of vocalizations required to activate the acoustical imprinting stimulus. Thus, this effect could simply reflect the fact that variable reinforcement regimens are usually more effective (hearing the imprinting stimulus likely has a reinforcement value for the chicks) [34].

Here, to avoid the perception of ‘repetitive’ or ‘mechanical’ movements in the social aggregation stimulus, we constantly varied the agents' trajectories across the eight scenes that composed it. Moreover, we did not implement perfect contingencies or perfect synchrony between the motion of the different kinds of agents. For instance, the chasers reacted to the movement of the forerunner with a delay, as expected in a naturalistic social interaction. However, we did not vary the temporal patterns underlying the scenes nor the duration of the delay in the agents' responses. Thus, chicks might have picked up the extreme regularity of the temporal contingencies between the motion of the different agents of the social aggregation display and turned their attention to the random display, in which these temporal contingencies were more variable. Some evidence obtained in infants suggests that behavioural variability could be an important cue for the recognition of animate agents capable of goal-directed behaviour [61], in line with our current results. Please note, however, that our chicks were not simply attracted by the mere presence of higher or lower temporal variability in the timing of the agents' movements. If that were the case, we should have observed a preference also in Experiment 2 and Experiment 3, in which only the forerunner or only the chasers were presented. In both these experiments, the agents' movements showed higher temporal variability in the random than in the social aggregation stimulus, but no preference was detected between them. Chicks seem to react selectively to the variability in the temporal contingencies between the motion of different agents (i.e. variability in the timing/predictability of the reaction of one agent to the behaviour of another agent). Thus, here we are not dealing with a simple preference for a more variable visual stimulus. Rather, we found a preference for a higher variability in a cue that is crucial for the detection of social interactions (temporal contingency between the motion of different agents).

Some evidence indicates that very young infants show an initial preference for perfect spatio-temporal contingencies between the behaviour of different objects (e.g. objects moving in perfect synchrony with the baby's own movements). In the immature infants, this might be instrumental for the development of visual recognition of their own body. However, during the first months of life, infants develop a preference for the imperfect temporal contingencies that resemble naturalistic social interactions, in which one animate agent will necessarily respond with a delay to the behaviour of its interaction partner (e.g. [58,62,63]; see also [59,64,65]). Indeed, often human adults and older infants show a preference for ‘independent/random displays' over chasing, while younger infants prefer chasing [42,44,45]. In Rochat *et al*. [42] adults reported that the ‘independent event was more interesting as it challenged participants' propensity to detect invariant relations in the dynamics of the two discs'.

As we mentioned above, our stimuli did not present perfect contingencies or synchrony between the motion of the different kinds of agents. For instance, the chasers reacted to the movement of the forerunner with a certain delay, as expected in a naturalistic social interaction. Moreover, none of the agents perfectly replicated the trajectory of another object as in Takahashi & Watanabe [60]. Even the chasers (whose movements were synchronized with each other) did not follow identical trajectories. However, the delays between the motions of the different agents were not variable between the eight scenes that comprised our ‘social aggregation’ stimulus, as it would probably be in a more naturalistic social interaction. Moreover, the general sequence of ‘behaviours’ displayed by the agents was also highly repetitive, which might have compromised animacy perception. Alternatively, the social aggregation stimuli might have been too easy to process for the chicks, which thus may have turned their attention towards making sense of the unpredictable interactions of the random stimuli's agents.

One could hypothesize that chicks behave similarly to older infants/adults in this regard, displaying a preference for the less predictable kind of interaction. The effect we found might thus be related to the fact that domestic chicks are a precocial species characterized by fast maturation. Although our chicks were visually naive, at the level of mere maturation, they are clearly not comparable with young human infants (e.g. chicks can already walk and feed in the first days after hatching). Thus, our results might be more in line with that reported in older infants, adults and (sometimes) in dogs (see above). Future studies could further investigate developmental effects. This can be done by testing chicks at different ages, to verify if the expression of this effect is limited to a specific post-natal period (and, if so, whether this time window is determined by the same hormonal mechanisms known for other social predispositions [18]). Moreover, since the processing of visual stimuli in chicks is highly lateralized [66–68], future studies could investigate whether the preference we observe here is also asymmetric (based on the literature, we would expect a major involvement of the left-eye system [68–71]).

Another interesting point is that our social aggregation stimuli contained some features of a ‘chasing’ display, which produces strong animacy perception in adults. Despite that, chicks clearly preferred the stimulus that did not contain this ‘chasing’ element. The attraction for variability in the temporal contingencies could be simply too strong and overcome any effect of chasing perception. However, this seems unlikely, since in Experiment 5 chicks were exposed to a control stimulus lacking this variability in the temporal contingency. Nevertheless, they did not show any preference for the social aggregation stimulus containing chasing sequences.

Another possibility is that some other aspects of our stimuli might have prevented the attraction to chasing. For instance, in human adults, the detection of chasing is impaired if the chasing behaviour is interrupted by long periods of random motion by the chaser [41]. Our social aggregation stimuli, indeed, included a phase in which the chasers started to explore the environment (when the forerunner was still). This was done to mimic a naturalistic filial interaction, in which chicks seek a balance between environmental exploration and maintaining brood aggregation. However, this could have impaired the perception of the chasing interaction (e.g. the behaviour of the chasers might appear non-goal directed, since after they joined the forerunner for a while, they then moved away from it). Future studies should test stimuli in which chasing is not interrupted by periods of random motion.

Another hypothesis could be that avian species are not sensitive to chasing displays, in line with the results of [50]. However, please note that also in this study, the ‘chasing’ stimulus differed from those typically employed in the human literature (the forerunners were moving randomly across the screen, rather than trying to escape the chaser). Future studies should investigate whether chicks, and avian species in general, show preference/higher sensitivity to prototypical chasing displays.

Overall, despite these open questions, our findings reveal that, prior to any visual experience, chicks can compute the temporal contingencies between the motion of different agents (an important cue for the recognition of social interactions) and use this to direct their approach towards the most promising social partners. This paves the way for future studies investigating the motion cues that naive organisms employ to detect different types of social interaction and, potentially, goal-directed behaviours.

## Data Availability

All the reported materials (data and stimuli) are freely available at https://doi.org/10.6084/m9.figshare.20347401 [72]. Supplementary material is available online [73].
